# Signs of Disease

**Published:** 1884-03

**Authors:** 


### SIGNS OF DISEASE.

1. If the brow is contracted, there is pain in the head ; if the head is hot, and turned restlessly from side to side, and the eyes stare, or there is a glare in them, there is inflammation, and

